# Contrasting Evolutionary Dynamics and Information Content of the Avian Mitochondrial Control Region and ND2 Gene

**DOI:** 10.1371/journal.pone.0046403

**Published:** 2012-10-05

**Authors:** F. Keith Barker, Mariah K. Benesh, Arion J. Vandergon, Scott M. Lanyon

**Affiliations:** 1 Bell Museum of Natural History, University of Minnesota, Saint Paul, Minnesota, United States of America; 2 Department of Ecology, Evolution and Behavior, University of Minnesota, Saint Paul, Minnesota, United States of America; Ben-Gurion University of the Negev, Israel

## Abstract

Mitochondrial DNA is an important tool for inference of population history in animals. A variety of mitochondrial loci have been sampled for this purpose, but many studies focus on the non-coding D-loop or control region (CR), which in at least some species appears hypermutable. Unfortunately, analyses of this region are sometimes complicated by segmental duplications, as well as by difficulties in sequencing through repeat expansions, driving many researchers to favor single-copy protein-coding or ribosomal RNA genes. Without systematic comparison, it is unclear if, how much, and what sort of information might be lost by focusing on coding regions, or conversely whether such regions might offer significant advantages over the CR. In this study, we compare the information content, both in terms of genealogy and tests of neutral equilibrium, of the mitochondrial CR and protein-coding ND2 gene of the red-winged blackbird (*Agelaius phoeniceus*) and its close relative the tricolored blackbird (*A. tricolor*). Both gene regions violate the standard infinite sites assumption central to moment-based population genetic inference, as well as exhibiting considerable among-site rate heterogeneity, obscuring significant departures from neutral equilibrium. Given the ubiquity of rate heterogeneity in mtDNA, use of more sophisticated tests that account for this should be obligatory. The two regions yield quite similar genealogical reconstructions, as well as indicating similar departures from neutral equilibrium assumptions for *A. phoeniceus*. However, individual Sanger-read-length fragments (∼600 bases) of the CR have significantly higher information content than comparable fragments of ND2, suggesting that limited sampling of the mitochondrial genome should focus on the CR.

## Introduction

Mitochondrial DNA (mtDNA) has proven a powerful tool for inferring population substructure and demographic history [1]–[3]. In particular, many studies of vertebrate mtDNA have focused on variation in the hypervariable domains of the mitochondrial control region (CR) [4]–[7]. Although it has proven extremely useful in some species, studies of the CR can be complicated by a number of factors. In many species, the CR contains one or more regions of repeat expansion (which may be heteroplasmic) that negatively impact PCR amplification, sequencing, or both [8]–[10]. In addition to this technical challenge, species in a number of animal groups exhibit segmental duplications involving the CR [11]–[13]. In some cases, this leads to creation of a pseudogenous region that may or may not be coamplified by PCR [14], [15], whereas in others two displacement-functional control regions appear to be maintained by concerted evolution [10], [16]–[18]. For these reasons, it is advisable if not obligatory for researchers studying the CR of previously unstudied species to establish the gene order and primary sequence of this and flanking regions prior to more extensive population sampling.

Consequently, many researchers have avoided sampling of the CR, instead focusing on regions encoding proteins or ribosomal RNAs [19]–[22]. Unfortunately, few studies have addressed the costs and benefits of using coding regions for population genetic inference in lieu of the CR, in terms of inferential power (but see [23]–[25]. For example, it is well documented that protein-coding genes of the mtDNA experience purifying selection (e.g., [26], [27]), which although it may not significantly bias the genealogical patterns important in comparative phylogeography [27], [28], may still have an impact on demographic inference. It is possible that in avoiding CR, researchers have abandoned a valuable source of information about evolutionary patterns and processes. Here, we investigate these questions by comparing genealogical and population genetic inferences obtained using samples from the CR and mitochondrial NADH dehydrogenase subunit 2 (ND2, a protein-coding gene commonly used in avian population-level studies; [29]–[31]) gene of the red-winged blackbird (*Agelaius phoeniceus*).

Inference of population processes from molecular data is assumption-laden, and it has become increasingly clear that the complexity of evolutionary dynamics in mitochondrial DNA, and in particular of the CR, has significant implications for such inference. For example, studies of human CR variation have established that this region violates many standard population genetic assumptions, and have led to increasingly sophisticated understanding of the impact of factors such as transition/transversion biases [32], [33], among-site rate heterogeneity [34], and among-lineage rate heterogeneity [35]. Theoretical and empirical studies have established that such complexity cannot be ignored, as it can bias point estimates of critical parameters such as genealogy, effective population size (*N_e_*) and time to common ancestry (*t_MRCA_*), as well as mislead tests of neutral equilibrium conditions (such as Tajima's *D* and Fu's *F_s_*; [36], [37]) which can lead to misinterpretation of population history [38]–[44]. Some progress has been made in developing methods that incorporate this complexity [33], [42], [45]–[48]. Even so, the general relevance of the standard assumptions for species other than humans, and the impact of using methods that incorporate violations of those assumptions on population genetic inference, has not been widely addressed. In our analyses, we apply a broad array of summary statistic, likelihood and Bayesian methods to evaluate the importance of realistic analytical assumptions in inference from CR and ND2 data.

The red-winged blackbird (*Agelaius phoeniceus*) is one of the most common bird species in North America, and has a wide distribution ranging from Costa Rica north through Alaska and Canada. Two studies of genetic polymorphism and population differentiation have been conducted on this species, one focusing on variation in mitochondrial DNA by a restriction fragment length polymorphism (RFLP) survey of populations in North America [49], and a second based on allozyme variation among populations in the U.S.A. [50]. These studies indicated that while polymorphism existed, there was little population differentiation in either mtDNA or nuclear protein-coding loci within the species. Given the current ubiquity of the species, these data were argued to be consistent with a recent population expansion. Subsequent reanalysis of the mtDNA data suggested that a northward trend of decreasing polymorphism within *A. phoeniceus* populations was consistent with expansion from a southern glacial refugium [51]. Neither of these analyses explicitly tested for neutral behavior of the markers considered. Likewise, neither study considered polymorphism within or divergence from the closely related sister taxon of this species (the California endemic tricolored blackbird *A. tricolor*), precluding rooting of intraspecific mtDNA genealogies and tests of lineage-specific rate constancy [52], [53]. In this study, we analyze CR and ND2 sequences from multiple populations of *A. phoeniceus* and *A. tricolor*, explore the complexity of inferred evolutionary dynamics at these loci within these species, test hypotheses regarding their evolutionary history in the context of this complexity, and investigate the relative information content of these two regions of the mitochondrial genome.

## Materials and Methods

### Samples and Sequencing

We obtained 31 samples of the red-winged blackbird *Agelaius phoeniceus*, and 10 samples of the closely related *Agelaius tricolor* as loans from multiple institutions (Appendix S1). Several samples were CsCl-purified mtDNAs derived from the study of Ball *et al*. [49], donated by J.C. Avise (University of California, Irvine); in addition, a purified mtDNA of the more distantly-related bronzed cowbird *Molothrus aeneus* provided by Avise served as an outgroup for this study. For tissue samples, whole genomic DNA was isolated using a QiaAmp DNA extraction kit (Qiagen, Valencia, CA). From each individual, we PCR amplified the complete mitochondrial control region using primers L16743 and H1248 [54] using 25 µL reactions with 0.625 U Qiagen HotStarTaq, standard buffer with 0.15 mM final concentration MgCl_2_, 400 µM of each dNTP, and 0.4 µM of each primer. Each reaction was subjected to an initial denaturation of 95°C for 15 min., followed by five cycles of 20 sec. denaturation at 95°C, 20 sec. annealing at 58°C, and 75 sec. extension at 72°C, then five more cycles each with annealing temperatures of 56 and 54°C, then 20 cycles with annealing of 52°C, and a final extension of 3 minutes. Products were resolved by electrophoresis on a 1% TAE NuSieve GTG agarose gel (Cambrex, Rockland, ME), excised, and melted in 200 µL ddH_2_0. Using the excised band as a template, the control region was reamplified in three segments using primer pairs L16743/H417, LCR3e/Emb1, and F304e/H1248 (Table 1 in [54]). Secondary amplifications were performed as were primaries, except that Qiagen Taq was used, and the thermal profile was 3 min. at 95°C, followed by 35 cycles of 20 sec. at 95°C, 20 sec. at 52°C, and 40 sec. at 72°C, and a final 3 min. at 72°C. The control region of both *Agelaius* species was characterized by poly-pyrimidine stretches in both the 5′ and 3′ hypervariable regions, which proved difficult to sequence in many individuals. To overcome this problem, we designed forward and reverse anchored primers for both poly-pyrimidine regions, which stretched across each region and were anchored by 5–16 bases of known flanking sequence (Table 1). Amplifications of ND2 were generally as for CR, except using primers L5215 and H1064 [55], [56] followed by sequencing with these and two internal primers (L5758-emb and H5766-emb [57]). All products were sequenced with BigDye v3.1 (Applied Biosystems, Foster City, CA), and electrophoresed on an ABI 3700 automated sequencer. Sequences were assembled into contig alignments using Sequencher v4.2 (GeneCodes, Ann Arbor, MI), and intraspecific samples were aligned by hand using MacClade v4.03 [58].

**Table 1:** Sequences and locations of novel control region primers used in this study.

Primer	Sequence	Position
LCR3e^1^	TCC AAC AGC CTT CAA GAA CA	L409
H886	AAT ATG TCC GGC AAC CAT TAC A	H886
F304e^2^	CTT GGC ACT GAT GCA CTT TG	L838
polyC-for	CCC CCC CCA GTA CAT TT	NA^3^
polyC-rev	GGG GGG GGT GGA GTG A	NA^3^
polyT-for	TTT TTA TTT TTT TTT ATC AAA CAA TAA AAC C	NA^3^
polyT-rev	AAA AAA AAA AAA TGA TGC GTA AAA	NA^3^

Position for conserved primers is given with reference to the complete *Vidua* mitochondrial genome (AF090341 [14]), and references are given for sequences that are modified versions of previously reported primers. The polyY primers were used for some individuals to obtain sequence adjacent to 5′ and 3′ poly-pyrimidine stretches.

1 modified from [54].

2 modified from [4].

3 matches region not identifiable in *Vidua*.

### Sequence analyses

Using Clustal X [59], [60] with default parameter values, putative control region sequences obtained from *Agelaius phoeniceus*, *A. tricolor*, and *M. aeneus* were aligned with complete or near-complete control region sequences from the estrildine finch *Vidua chalybeata* (AF090341 [14]), and more closely related members of the avian family Fringillidae, including *Fringilla coelebs* (U76250), *Carduelis chloris* (U56075), *Emberiza schoeniclus* (AJ243929), and *Geospiza scandens* (AF109813). Based on this alignment, conserved CR motifs were identified by comparison with previous studies [4], [61], [62], to establish homology and successful amplification of the targeted region. In addition, flanking sequences of the genes for tRNA-Glu and tRNA-Phe were compared with data from the complete mtDNA genome sequence of *Vidua*. Finally, primarily as a check for errors in PCR or contig assembly (see [63]), we applied six tests for detecting recombination to a combined matrix of unique haplotypes from all three species studied, using RDP v2.0 [64].

For each locus, the genealogy of unique haplotypes was inferred using maximum likelihood (ML) and Bayesian methods. Since vertebrate mitochondrial DNA does not appear to recombine (but see [63]) the two regions sampled here are expected to share their underlying genealogy. However, since a primary objective of this study was to evaluate their relative information content, phylogenetic trees were inferred separately for the two regions. Model selection was performed using the Akaike information criterion (AIC), as implemented in ModelTest v3.7 [65]. In addition, we tested whether a molecular clock fit the data. To evaluate the uniformity of evolutionary process across each locus, parameters under the best fit model were evaluated in a sliding window of 250 bases in length, starting with the first base and moving 1 base at a time across the alignments. Subsequent to model selection, the maximum likelihood trees of haplotype relationships were inferred by heuristic searches using PAUP* v4.0b10 [66] under the best-fit model and initial parameter estimates (10 random addition sequence replicates, tree bisection and reconnection [TBR] branch swapping, branches of effectively zero length collapsed). Support for relationships in the trees was estimated by the non-parametric bootstrap [67] (200 replicates of TBR branch swapping on initial trees obtained by neighbor-joining with ML distances). Bayesian analyses under best-fit models were performed using MrBayes v3.2 [68], using default priors, performing paired simultaneous runs each with parallel implementation of Metropolis coupling with one unheated and three heated chains [69] (default temperature value). Multiple runs were performed, among run variance examined quantitatively within each, and convergence of nodal posterior probabilities and parameter values across runs was evaluated qualitatively. Given the trees obtained in these analyses, site-specific patterns of change and regional variation in evolutionary rates were estimated using ML and empirical Bayes methods [70] as implemented in PAML v3.12 [71].

Summary statistics of DNA polymorphism were calculated for *A. phoeniceus*, *A. tricolor*, and individual populations of the former species using DnaSP v4.0 [72], and R scripts (R Development Core Team 2011) written by the authors. Statistics reported include the number of haplotypes (H), haplotype diversity *h* [73], nucleotide diversity π [74], number of segregating sites (S), and the per site version of Watterson's estimator of the neutral parameter 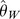 [75]. We also conducted single-locus tests of neutral equilibrium assumptions using Tajima's *D* [36] and Fu's *F_s_* [37], comparing values of these calculated from the data to the standard expectation from the β distribution (in the case of *D*), and to null distributions obtained via infinite sites coalescent simulations conditioning on 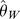 (for both statistics). It has been noted that both *D* and *F_s_* are sensitive to among-site rate heterogeneity, with the former yielding excessively positive values [39], [42], and the latter significantly negative values, even under neutral equilibrium conditions [41]. Therefore, we also compared these statistics to null distributions calculated with the batch processing facility of Arlequin 3.1 [76] from data sets simulated under the best fit model of sequence evolution and coalescent parameters estimated from the data, using Treevolve 1.3.2 [77].

In addition, we calculated estimates of the neutral parameter correcting both for violations of the infinite sites assumption, and for the assumption of uniform rates among sites, as described previously [42], [47]. These statistics include 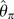, 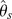, and 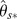, deriving from the average pairwise number of mutations between haplotypes in the sample, the number of segregating sites, and the minimum number of mutations (for each site, the number of bases present at that site minus 1) respectively. These statistics depend on an estimate of the overdispersion parameter (α parameter of the Γ distribution of among-site rate heterogeneity) for the region of interest: the values used were those estimated during maximum likelihood genealogical analyses (see above). Misawa and Tajima [42] also developed finite-sites statistics for evaluating the validity of neutral equilibrium assumptions, incorporating among-site rate heterogeneity, analogous to Tajima's *D*. Values of their 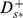 (correcting an error in their published equation; see Appendix S2) were calculated and compared to confidence limits on *D* given in Table 2 of Tajima [36], as well as to null distributions based on the simulated data used in evaluation of *D* and *F_s_* above.

**Table 2:** Summary of polymorphism data and population parameter estimates in populations of *Agelaius phoeniceus* and *A. tricolor*.

Species	Gene	L	n	*k*	*h*	S	η	I	π	*θ_W_*	*θ_π_*	θ_s*_
*phoeniceus*	CR	1208	31	25	0.980 (0.010)	65	67	1	0.0090 (0.0014)	0.0135 (0.0044)	0.0102 (0.0048)	0.0165 (0.0053)
	ND2	1041	31	17	0.920 (0.001)	55	55	0	0.0077 (0.0014)	0.0132 (0.0044)	0.0081 (0.0038)	0.0142 (0.0046)
*tricolor*	CR	1206	10	8	0.930 (0.080)	12	12	0	0.0032 (0.0005)	0.0035 (0.0017)	0.0034 (0.0018)	0.0037 (0.0018)
	ND2	1041	10	3	0.689 (0.011)	3	3	0	0.0011 (0.0003)	0.0010 (0.0007)	0.0011 (0.0008)	0.0010 (0.0007)

L = sequence length, n = number of individuals sampled, *k* = number of unique haplotypes, *h* = haplotype diversity (s.d. in parentheses here and subsequently), S = number of segregating sites, η = minimum number of mutations ( = S* of [42]), I = number of segregating indel mutations, π = nucleotide diversity [73], *θ_W_* = Watterson's estimator [73], *θ_π_* = Misawa and Tajima's estimator (eqs. 5a and 6a in [42]), θ_s*_ = Misawa and Tajima's estimator (eqs. 5c and 6c in [42]). All calculations of Misawa and Tajima's estimators and test used an α = 0.102 for CR, and α = 0.279 for ND2 (see text, Figure 2).

While some of the above statistics begin to incorporate complex evolutionary dynamics, none considers the full likelihood of the data, including genealogical relationships among haplotypes. Using LAMARC v2.1.5 [78] and BEAST v1.4.8 [79], we estimated the neutral parameter for each species using coalescent-based Markov chain Monte Carlo likelihood and Bayesian analyses, incorporating biases in transition and transversion probabilities, uneven nucleotide frequencies, and among-site rate heterogeneity. In addition to the evaluation under equilibrium assumptions, we explored one common violation of neutral equilibrium expectations by allowing for the possibility of population growth [45], [46]. In likelihood analyses with LAMARC, for each species, substitution model, and parameter set (i.e. both with and without population growth), we ran 10 short chains of 10,000 steps, followed by two long chains of 2·10^6^ steps each, with 1000 burn-in steps per chain, and in both cases thinning the chains by sampling every 20^th^ state. In every case, we employed heating with three chains (temperatures of 1, 1.2 and 1.4), and ran at least two replicates per analysis to evaluate consistency of parameter estimates. Approximate confidence intervals for parameters in these models were estimated by percentage profile likelihoods. In Bayesian analyses with BEAST, for each species we analyzed each data set with its optimal model of sequence evolution, both without and with exponential population growth, running chains of 1·10^7^ generations and using default priors (excepting analyses of ND2 including growth, which used an exponential prior, see Results).

## Results

### Sequence characteristics

All sequences have been submitted to GenBank (accessions JX512560-JX512643). Amplification of the complete CR yielded single, clear products that sequenced cleanly except near two polypyrimidine stretches. Complete mitogenome sequencing of *Agelaius* and *Molothrus* (FKB, pers. obs.) confirms that these taxa share the “ancestral avian” configuration with no CR duplication [12]. Alignment of CR sequences from the three species studied here with available control region sequences from the literature allowed identification of conserved sequence motifs, including the F, D, C, “bird similarity” and B boxes, as well as the conserved sequence block 1 (CSB-1; [4], [61], [62]). Full length sequences from *Agelaius phoeniceus* were 1207 or 1208 bases (due to a single base indel polymorphism), including 35 bases of tRNA at the 5′ end and 19 bases at the 3′, and excluding 53 bases in the poly-pyrimidine regions. Corresponding sequences of *Agelaius tricolor* were 1206 bases in length, while the single haplotype of *Molothrus aeneus* was 1193 bases. Alignment of sequences from these three species yielded a data set of 1210 positions. We found no evidence for recombination in these data (results not shown). Sequences of ND2 obtained were all 1041 bp in length, and exhibited an open reading frame under the vertebrate mitochondrial code, with a single stop codon at the end of the alignment, as expected for functional sequence.

### Model fitting and tree estimation

**Figure 1:**
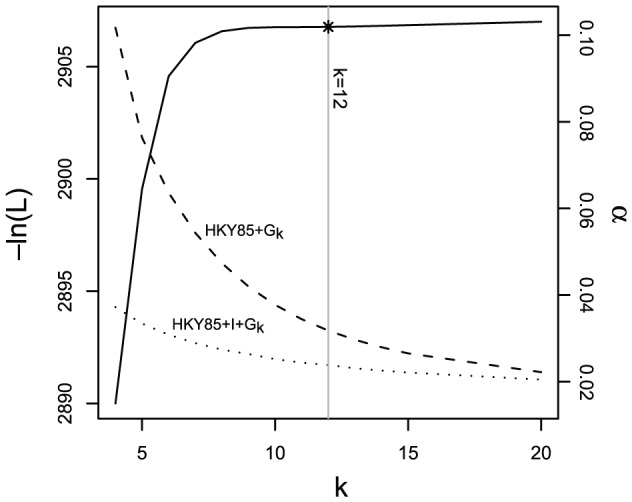
Number of rate categories (k) affects model likelihoods and parameter estimates. Discrete Γ model negative log-likelihoods for the control region data are shown on the left axis, and estimated α parameters on the right. The line at k = 12 indicates the point at which addition of a class of constant characters (p_iv_>0) does not significantly improve the model fit.

Maximum likelihood model fitting to the set of unique control region sequences from both *Agelaius* species and the outgroup suggested the HKY85+I+G_4_ ([80]; the subscript 4 indicates a four category discrete Γ approximation) model of sequence evolution as the best fit by the AIC. However, further exploration indicated that this result was critically dependent on the number of categories used to approximate the Γ distribution. As categories were added, the significance of the difference between the HKY85+I+G_n_ and HKY85+G_n_ model decreased, until at 12 categories, the two were indistinguishable (Figure 1). Over this same range, estimates of α in the HKY85+G_n_ model went from an extreme low of 0.02, up to a plateau around 0.102 (although additional categories continued to increment this estimate slightly). For this reason, and to simplify direct comparisons with results obtained from PAML, we selected the HKY85+G_12_ model as the best fit to these data. By contrast, analysis of the ND2 data yielded strong support for the GTR+I model [80], [81] of sequence evolution. Addition of categories to the discrete Γ approximation actually reduced the likelihood of these data (result not shown), suggesting that preference of the invariant sites model is not an artifact. We were not able to reject the hypothesis of rate constancy among lineages for CR (−2 ln[L] = 41.6, df = 32, p = 0.12) or for ND2 (−2 ln[L] = 24.5, df = 19, p = 0.18), suggesting clock-like evolution among haplotypes of both loci for the three species sampled.

**Figure 2:**
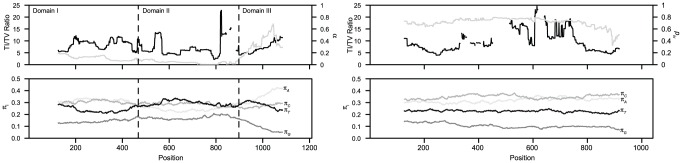
Sliding window analysis of model fit across *Agelaius* control region and ND2 regions. Each point plots parameter values of either an HKY85+G_12_ (for CR, left), or HKY85+I (for ND2, right) model, for a 250 base pair window centered at that position, for each position sampled (step size = 1). The upper panels show values of the transition/transversion ratio (left axis and black line; some spurious values estimated due to the absence of transversions in given windows omitted), and the parameter of the discrete Γ approximation (CR; 12 categories) or proportion of invariant sites (ND2; right axes and gray lines). The lower panels show the nucleotide proportion parameters π_i_.

A sliding window analysis of model parameters across alignments of the CR suggests substantial heterogeneity in some parameters (Figure 2), reflecting the “domain” structure previously observed in avian mitochondrial genomes [4], [62]. Among the most notable features are the extremely high among-site rate heterogeneity in domain II, much lower rate heterogeneity in domain III, and distinctly low frequency of G and high frequency of A in domain III. By contrast, estimated parameters for ND2 are quite uniform across the gene (Figure 2). Values of the transition/transversion ratio vary significantly, mostly because of the very small number of transversions in the middle portion of the gene, and the proportion of invariant sites (*p_iv_*) declines abruptly at the 3′ end. In addition, the equilibrium frequency of guanine residues appears to decline slowly across the gene, compensated primarily by increases in adenine and cytosine, but the magnitude of these differences is quite small. Generally, the CR exhibits much more regional variability in evolutionary dynamics than ND2.

**Figure 3:**
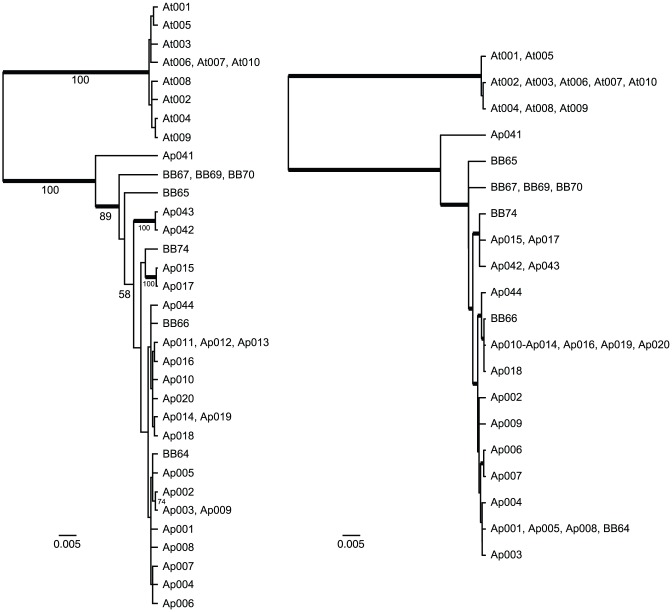
Trees obtained in maximum likelihood analyses of CR and ND2 data. Shown are: A) one of ≥270 maximum likelihood trees obtained via heuristic search under the HKY85+G_12_ model (−lnL = 2907.7, κ = 16.702, π_A_ = 0.312, π_C_ = 0.285, π_G_ = 0.134, α = 0.1019) with a molecular clock, for the set of unique *Agelaius* CR haplotypes and a single outgroup *Molothrus* (see Appendix S1 for sample identification); and B) the single best tree obtained via heuristic search under the GTR+I model (−lnL = 2337.7, r_AC_ = 5.122, r_AG_ = 118.9, r_AT_ = 3.560, r_CG_ = 8.226e−4, r_CT_ = 47.54, π_A_ = 0.310, π_C_ = 0.355, π_G_ = 0.098, *p_inv_* = 0.723) with a molecular clock, for the unique ND2 haplotypes and the same outgroup. Numbers next to branches indicate support as estimated by the non-parametric bootstrap (200 replicates; molecular clock not enforced due to time constraints), and thickened branches indicate estimated Bayesian posterior probabilities ≥0.95.

Maximum likelihood heuristic searches for the CR data, assuming the HKY85+G_12_ model with rate constancy, recovered at least 270 equally-likely trees for the set of unique haplotypes, prior to abortion of the search at ten days (Figure 3). Overall, support for most nodes in these trees was low, as many closely related but distinct haplotypes were sampled. The most strongly supported branches in the tree were those defining monophyly of *Agelaius phoeniceus* and *A. tricolor* (100%), and a basal branch within *A. phoeniceus*, separating the single Nicaraguan haplotype (Ap041) from all others sampled (89%). Two other interior nodes received moderate support (58 and 74%), and two closely-related pairs of haplotypes were clustered together in all replicates (Figure 3). Bayesian analysis of the data under the same model yielded a consensus topology and support values quite similar to those of the ML trees. Although the ND2 sample distinguished fewer haplotypes than the CR (17 versus 25, Table 2), genealogical analysis of the data yielded broadly congruent results. Maximum likelihood analysis of the ND2 data yielded 9 equally-likely trees, whose consensus was well resolved, with only two polytomies of three haplotypes apiece. Notably, the ND2 data appeared to resolve relationships among haplotypes more robustly than the control region, with 9/19 internal branches on the ND2 consensus recovered with ≥0.95 estimated posterior probability, compared to 5/32 for the CR. Given this difference in resolution and support, the two genealogies are congruent as expected, with the ND2 data recovering monophyly of the two *Agelaius* species, the basal split within *A. phoeniceus*, and the separation of two Mexican haplotypes (BB65 and BB67/69/70) as sequential sister lineages to the remaining variation.

### Polymorphism and tests of neutral equilibrium

Populations of both species of *Agelaius* harbored appreciable sequence diversity at the mitochondrial control region (Table 2), with relatively lower amounts of variation at ND2. As noted above, nearly every CR haplotype obtained from either species was unique, and the average uncorrected divergences among haplotypes were 0.9% and 0.3% for *Agelaius phoeniceus* and *A. tricolor* respectively (Table 2). By contrast, ND2 exhibited fewer unique haplotypes, and divergence among those haplotypes was reduced in both species (0.3% and 0.1%). As expected given their relative census population sizes (see Discussion), polymorphism was substantially higher in *A. phoeniceus* than in *A. tricolor*, both in nucleotide diversity and in neutral parameter estimates based on segregating sites (Table 2). Divergence between the two species at the CR was substantial, with 37 fixed nucleotide differences and two fixed length differences, and an average uncorrected haplotype divergence of 5.0%. Despite the fact that the CR is more polymorphic, ND2 exhibits more differentiation, with 61 fixed differences between the two species, and 7.7% average uncorrected haplotype divergence, similar to that observed at other protein-coding loci such as cytochrome *b* (7.0% [82]). This reflects the more consistent evolutionary rate across the protein-coding gene, which lacks an invariant region analogous to Domain II of the CR (see below), and experiences reduced saturational effects (see below).

**Figure 4:**
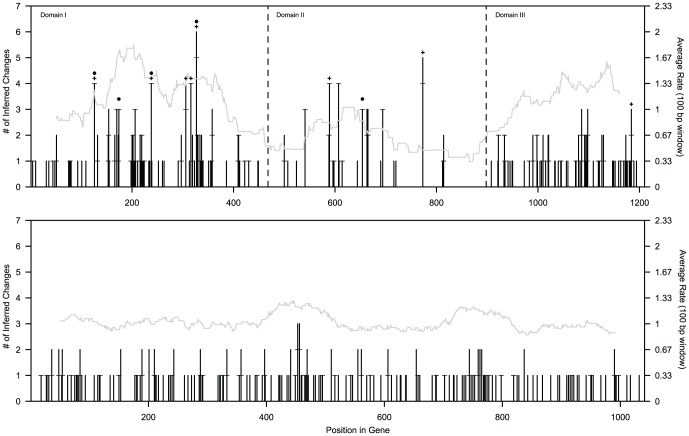
Rate heterogeneity in *Agelaius* control region and ND2. Shown are the inferred number of changes per site (left axis and black line; based on marginal reconstruction of ancestral states on arbitrarily-chosen ML trees) in the alignment of *Agelaius* CR (above) and ND2 (below), and sliding window analysis (plotted at the center of each window, width = 100 bases, step = 1 base) of site-specific rate estimates (right axis and gray line; empirical Bayes estimates from PAML 4.4, correlation of true and estimated rates was 0.583 for CR and 0.545 for ND2). Asterisks mark five hotspots identified by polymorphism within both *A. phoeniceus* and *A. tricolor*, and plus symbols mark “hypervariable” CR sites (defined as sites in the 95^th^ percentile or higher of site-specific rate estimates for variable sites). Horizontal lines on bars for individual site change reconstructions indicate the number of intraspecific mutations as opposed to fixed differences between species.

As noted above, substantial rate heterogeneity exists among portions of the CR. Consistent with many avian and mammalian species [82], [83], most polymorphism was at sites in domains I and III of the control region, with only 13/65 *A. phoeniceus* and 2/12 *A. tricolor* polymorphisms segregating in domain II, and a higher proportion of polymorphism in domain I than in domain III (∼2∶1). Even without phylogenetic analysis, two sites in domain I segregate three different nucleotides each, demonstrating the occurrence of multiple mutations per site (compare S and η, Table 2). Phylogenetic analyses of these data suggest that many sites segregating only two nucleotides have also experienced multiple mutations ranging from 2 to 4 changes per site, based on marginal maximum likelihood reconstructions (Figure 4). By contrast, the ND2 data exhibit more uniform evolutionary rates and have no sites segregating more than two bases. Similarly to the CR data, phylogenetic analysis reveals many more mutations than estimated based on the number of segregating sites, but all sites with more than one mutation have only two inferred changes within a species (Figure 4). These results demonstrate that infinite sites models are inadequate representations of the evolutionary history of these data, and that analyses incorporating finite-sites approaches (especially those integrating genealogy) should be preferred.

The data for both species show evidence of violating neutral expectations. Given that the data clearly violate the assumptions of uniform evolutionary rates and infinite sites (see above), estimates of neutral parameters and tests of neutral assumptions should take these facts into account. We re-estimated both using the methods of Tajima [47] and Misawa and Tajima [42], as well as by comparing standard statistics (*D* and *F_S_*) to null distributions generated under finite sites models. Corrections of neutral parameter estimates and finite sites simulations require an estimate of the α parameter of the Γ distribution describing rate variation among sites (the second parameter of the distribution β is chosen by default such that the expectation of the distribution is 1). For this purpose, we used values of α = 0.102 for the CR and α = 0.279 for ND2, estimated by ML under the HKY85+G_12_ model (note that this was not the preferred model for ND2, but statistical corrections for invariant sites have not been developed). Incorporation of a finite sites model yielded substantially higher estimates of neutral parameters for both species, ranging from a 4% increase for the parameter estimates of *A. tricolor*, up to a 22% increase for the segregating sites estimate for *A. phoeniceus* (Table 2). Nearly all moment-based tests of neutral equilibrium conditions for both CR and ND2 of *A. phoeniceus* (Table 3) were significantly different from their null distributions based on both infinite and finite sites assumptions (*F_S_*), or finite sites expectations alone (*D*, 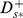): the only non-significant test was *F_S_* for ND2. In all cases, significant statistics were consistent either with a selective sweep or population expansion (i.e. negative values [36], [37], [42]). By contrast, no summary statistic test of neutral equilibrium in data from *A. tricolor* reached significance (Table 3).

**Table 3:** Tests of neutral equilibrium assumptions in *Agelaius phoeniceus* and *A. tricolor*.

Species	Gene	*D*	95% CI (β)	95% CI (FS)	D_s*_^+^	95% CI (FS)	*F_s_*	5^th^ Percentile (FS)	5^th^ Percentile (IS)
*phoeniceus*	CR	**−1.327**	−1.807, 2.021	−1.274, 2.103	−1.822*	−1.805, 1.854	−9.370**	−6.849	−6.016
	ND2	**−1.570**	−1.807, 2.021	−1.491, 2.023	−2.013*	−1.578, 1.970	−2.387	−24.787	−5.887
*tricolor*	CR	−0.376	−1.733, 1.975	−1.730, 1.709	−0.512	−1.869, 1.686	−2.714	−16.519	−3.116
	ND2	0.398	−1.733, 1.975	−1.562, 1.844	0.280	−0.470, 2.667	0.800	−28.461	−2.096

Values of Tajima's *D* [36] are accompanied by 95% confidence intervals of the statistic based on the the expected β distribution under the infinite sites model (table 2 in [36]), and as assessed for data simulated under the best–fit finite-sites model of sequence evolution (FS; see text). Values of Misawa and Tajima's 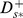 (modified; see Appendices 2 and 3) and of Fu's *F_s_* statistic [37] are also given, along with 95% CIs (or 5^th^ percentiles for *F_S_*, which is evaluated one-tailed) estimated by simulation as for *D*. Significance of *D*, D_s*_^+^, and *F_s_* given either the β distribution or coalescent simulations under the infinite sites model (*F_s_* only) is shown by asterisks (*P<0.05, **P<0.01), and values significant at the α = 0.05 level given finite sites simulation CIs are highlighted in bold.

We also performed likelihood and Bayesian evaluations of the neutral parameters for *A. phoeniceus* and *A. tricolor* as a whole, using a finite sites model with discrete rate categories, as implemented in LAMARC v2.1.3 [45], [46], [78] and BEAST v1.4.8 [79]. For the substitution parameters, we used the ML estimates for the HKY85 (CR; reparameterized for the F84 model as necessary [84]) and GTR (ND2) models obtained above, and for rates we used five (LAMARC) or 10 (BEAST) categories with α values given above (in LAMARC, we entered mean rates from a discretized Γ distribution as calculated from equation 10 in [85]). Likelihood and Bayesian non-equilibrium analyses of the data yielded nearly identical results (Table 4). The most notable difference was in estimates of *θ* for the *A. tricolor* CR data when allowing for exponential population growth, where likelihood values were more than twice those estimated by Bayesian methods, and the estimated confidence interval was ∼50 times larger. This result persisted in multiple runs, and modification of the sampling regime (additional replicates, more heated chains) had little effect. Estimates of *θ* obtained for each species (Table 4) were from 2 to 3 times those obtained from summary statistics, even those assuming a finite sites model with rate variation (Table 2). This is likely due to a large number of recurrent mutations at a few “hotspot” sites, as was apparent from examination of changes across the haplotype trees (see above and Discussion). Approximate 95% confidence intervals (estimated by profiling with LAMARC, see Methods) and 95% highest posterior density intervals (calculated in Tracer v1.4 from BEAST results [86]) for the growth parameter *g* excluded 0 in only one case, the CR data from *A. tricolor*, which suggested significant positive population growth in this species. Estimates of *g* from *A. phoeniceus* were consistently lower than for *A. tricolor* by about an order of magnitude, and only marginally significantly different from 0 for the CR, with a 90% credibility interval of (8, 211), while the ND2 data yielded similar *g* values but much wider confidence and credibility intervals (Table 4). In fact, in all cases the CR data yielded smaller confidence and credibility intervals on *g* than did the ND2 data (by a factor of 2–20). By contrast, the ND2 data generally yielded much smaller confidence and credibility intervals on *θ* than did the CR data (by a factor of 1.5–5), consistent with the expectation of increasing variance with greater haplotype divergences (e.g., the number of segregating sites *S* is approximately Poisson for large sample sizes, with variance equal to the mean [75]).

**Table 4:** Maximum likelihood and Bayesian estimates (approximate 95% confidence and credibility intervals in parentheses, parameter estimates for intervals exclusive of 0 are highlighted in bold) of the neutral parameter and population growth for *Agelaius phoeniceus* and *A. tricolor*, derived from analyses using LAMARC v2.1.3 [78] and BEAST v1.4.8 [79].

Species	Gene	*g*	*θ_ML_*	*g_ML_*	*θ_Bayes_*	*g_Bayes_*
*phoeniceus*	CR	= 0	0.0355 (0.0221, 0.0580)	0	0.0225 (0.0122, 0.0330)	0
		≠0	0.0454 (0.0254, 0.0848)	79 (−28, 214)	0.0312 (0.0149, 0.0529)	102 (−16, 229)
	ND2	= 0	0.0146 (0.0088, 0.0245)	0	0.0076 (0.0042, 0.0117)	0
		≠0	0.0162 (0.0089, 0.0306)	54 (−141, 247)	0.0089 (0.0041, 0.0152)	60 (−161, 265)
*tricolor*	CR	= 0	0.0070 (0.0029, 0.0175)	0	0.0048 (0.0015, 0.0097)	0
		≠0	0.0389 (0.0061, 1.5475)	**1728** (272, 7203)	0.0152 (0.0028, 0.0325)	**1688** (235, 3546)
	ND2	= 0	0.0010 (0.0002, 0.0038)	0	0.0006 (0.0000, 0.0015)	0
		≠0	0.0013 (0.0002, 0.0124)	783 (−4830, 20829)	0.0053 (0.0000, 0.0167)	19450 (−4076, 73939)

The growth parameter *g* was either fixed at 0 ( = 0) or allowed to vary (≠0).

## Discussion

### Sequence evolution in the control region and ND2

The pattern of sequence evolution in the *Agelaius* mitochondrial control region is similar in most respects to that observed in many other bird and mammal groups. Domains I and III contain the great majority of polymorphism, although some domain I sites appear to have higher site-specific rates, and domain III appears to have a higher proportion of variable sites evolving at lower rates (Figure 2). Transition bias is a prominent feature of the data: the global ML estimate of the transition/transversion rate of ∼8 is on par with results from other bird species [4], [61], [62]. However, we were unable to reject the hypothesis of equal A–G and C–T transition rates (−2 ln L = 0.01, p = 0.91), which have been shown to differ in human CR sequences [33]. In terms of nucleotide frequencies, the data exhibited the typical vertebrate mtDNA deficiency of light strand guanine, with a pronounced decrease in π_G_ in domain III, primarily compensated by increasing π_A_ (Figure 2), as has been observed in other avian taxa [87].

Perhaps the most notable feature of *Agelaius* CR evolution is the marked heterogeneity in evolutionary rate among sites. Overall, the ML estimate of α was 0.102, which is similar to estimates from human mtDNA (e.g., [33]), but substantially lower than values reported from other birds (e.g., [88]). Rarefaction analyses of human CR variation [89] suggest that samples of less than ∼20 haplotypes can yield extremely high or low values of α by chance, but estimates stabilize rapidly with increasing sample size. Given that we have sampled 41 *Agelaius* haplotypes (31 from *A. phoeniceus* and 10 from *A. tricolor*), and included an outgroup in parameter estimation, it is unlikely that our result is an artifact of sampling. Many studies have reported α from studies of interspecific variation [61], [62], [87], [90], [91], and these values are generally much higher than observed for *Agelaius* (7 comparisons, range = 0.09–1.63, 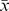 = 0.447). It should be noted however, that inclusion of more distantly related sequences seems to lead to increased estimates of α (e.g., [92]), possibly due to differing constraints or mutational pressures among taxa [93].

Rate heterogeneity itself varies across the CR. In a sliding-window analysis of model fit with HKY85+G_12_ parameterization, estimates of α ranged from essentially zero in portions of the central domain to a maximum in domain I of 0.194, and a global maximum of 0.687 for a single window in domain III (Figure 2). As most human sequencing has focused on the so-called hypervariable regions I and II (HVRI and II; domains I and III in this study), variation in rate heterogeneity across the entire control region has not been broadly examined. However, focusing on the two hypervariable regions, Meyer et al. (1999) found higher estimates of α in HVRI (0.26) than in HVRII (0.13), suggesting stronger rate heterogeneity in the latter region, in contrast to the pattern in *Agelaius*. Few studies have examined variation across the avian CR using comparable models (e.g., [87], [88], [90]), and these have yielded similar results in showing much higher rate heterogeneity within domain II than the flanking domains, and apparently higher rate heterogeneity in domain I than in domain III.

A notable feature of the control region of many organisms is the presence of mutational hotspots [34], [93], [94]. One criterion for identification of mutational hotspots is the occurrence of correlated polymorphisms—sites that are polymorphic in more than one species, despite fixed differences (and thus reciprocal monophyly) between them [93]. Five sites in the CR alignment (126, 174, 238, 327, and 654) were polymorphic in both *A. phoeniceus* and *A. tricolor*. Strikingly, one of those sites (327) was also segregating three variants (A, G, and T) within *A. phoeniceus* alone. In addition, we discovered a number of sites in the CR that appear to have experienced between two and four mutations within *A. phoeniceus* alone (Figure 4). Eight sites (126, 238, 306, 316, 327, 589, 773, and 1184) comprise the top 5% of estimated rates, three of which are also polymorphic in both *Agelaius* species. Thus, a total of ten control region sites might reasonably be termed hotspots. Of these, six are within domain I, three within domain II, and only one within domain III.

By contrast, the ND2 data are quite uniform in evolutionary terms, showing consistent base composition, average mutation rates, and rate heterogeneity (as measured by *p_iv_*) across the entire gene (Figures 2, 4). Transition/transversion rate ratios vary substantially (Figure 2), but this is primarily due to the complete or near-complete absence of transversions in some windows analyzed. The most notable local variation in evolutionary dynamics is the reduced *p_iv_* at the 3′ end of the gene (Figure 2). There was no indication of mutational hotspots in ND2, as the largest number of mutations per site within a species was two (compared to four in the CR), and there was no site variable in both *A. phoeniceus* and *A. tricolor*.

### Finite sites and parameter estimation

Our results clearly demonstrate that *Agelaius* mitochondrial DNA sequences violate several standard assumptions of population genetic models. Most notably, both regions sampled here exhibited significant among-site rate heterogeneity, reaching extremes in the CR. In addition, it is apparent that both loci—but in particular the CR sequences of *A. phoeniceus*—have experienced multiple mutations per site (Figure 4), violating the infinite sites assumption that lies at the heart of most standard polymorphism statistics and tests of neutral equilibrium. Ignoring violations of model assumptions can seriously bias inferences of important population genetic parameters (e.g., [38], [42], [92]). Although the global importance of this bias might be dismissed on the grounds that the CR is a single genetic locus [95], analyses of mtDNA remain an important tool in genetic studies of natural populations, and the data should be examined with the most realistic models.

It is clear that violation of infinite sites and uniform rates assumptions has a significant impact on polymorphism estimates within blackbirds, with estimates that relax them being generally higher, sometimes substantially so (Table 2). Perhaps more importantly, tests of neutral equilibrium are severely compromised by failure to account for among-site rate heterogeneity. In this empirical example—as in simulated data—uncorrected values of Tajima's *D* fail to reject the null hypothesis of neutral equilibrium [42]. Specifically, Tajima's *D* yielded non-significant values for both species of *Agelaius* when compared to its standard β distribution or a null distribution based on infinite sites assumptions (Table 3). By contrast, comparison of *D* for *A. phoeniceus* as a whole to a null distribution based on a finite sites model indicated significance with both CR and ND2 data. Using a corrected *D* (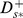) compared to a standard β distribution likewise yielded significant values. Values of Fu's *F_s_* compared to a standard null appear to have the opposite problem of an excessive Type I error rate [42], which is reflected in critical values for this statistic simulated here (Table 3). However, this bias does not affect interpretation of the *Agelaius* data, where three out of four values are insignificant with both infinite and finite sites null distributions, and the fourth (for *A. phoeniceus* CR) is significant relative to both null distributions. Thus, use of summary statistics taking evolutionary dynamics—in particular rate heterogeneity—into account yields generally consistent results. Given the ubiquity of these dynamics in animal mtDNA (notably, ND2 is among the *least* rate-heterogeneous genes in the blackbird mitogenome; FKB pers. obs.), wider application of these methods should be considered obligatory. The simplicity of calculating 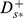 once an estimate of α has been obtained is appealing, and this may be an acceptable corrective, although the apparent increased power of *F_s_* to detect demographic expansion or selective sweeps [37] may be sacrificed. However, additional power analyses of the latter test with rate heterogeneity should be performed to ascertain that this advantage still pertains under such conditions. Sampling of one or more outgroups should generally aid in estimation of α [96], so this should be considered standard practice in phylogeographic and population genetic studies, especially in taxa with relatively low intraspecific polymorphism, where α may be particularly difficult to estimate.

A more sophisticated approach to incorporating model complexity is possible with likelihood and Bayesian analyses that integrate demographic parameter estimates over genealogies of the sampled loci. Interestingly, when applied to the mitochondrial data sampled from these species of *Agelaius*, these more sophisticated methods yield conclusions essentially opposite to those obtained from summary statistic analyses (Table 4). As discussed above, the latter analyses point to either a selective sweep or demographic expansion within *A. phoeniceus* but not *A. tricolor*, whereas the former analyses suggest relative stability for *A. phoeniceus* and population expansion for *A. tricolor*. This is overstating the case somewhat, as the analyses incorporating exponential growth for *A. phoeniceus* only just fail to exclude zero population growth at the 95% probability level, and are significant at the 90% level. Likewise, the evidence for expansion of *A. tricolor* derives only from CR, and not ND2 (although the estimates from both gene regions are consistent). However, the estimated growth rates for *A. phoeniceus* are approximately two orders of magnitude lower than those estimated for *A. tricolor* (excluding the extremely high value inferred in the Bayesian analysis of ND2), suggesting a contrast in demographic history (or selective effects) that is not hinted at in summary statistic analyses, even those incorporating rate heterogeneity (Table 2). Overall, the trend with increasing model complexity appears to be of greater sensitivity to violations of neutral equilibrium conditions in both species.

### Population dynamics versus selection in Agelaius

Although the sampling of individuals in this study is limited due to our focus on the contrasting evolutionary dynamics of mtDNA data gene regions, these data offer insight into the demography and evolution of the two species studied. Previous studies of mtDNA variation in *A. phoeniceus* found relatively low levels of polymorphism (π_RFLP_ = 0.002 [49]). Using a “universal” avian substitution rate of mitochondrial evolution (2% pairwise divergence per million years [97], see also [98], [99]) as an estimate of the mutation rate, and assuming a generation time for females of *T* = 1.63 (based on age at first breeding data in [100]), an estimate of the mutation rate across the mtDNA genome is μ = (1.63 years/generation)×(0.02·10^−6^ mutations/haplotype pair/year)÷(2 haplotypes/haplotype pair) = 1.63·10^−8^ mutations/lineage/generation. Given the neutral expectation that π = 2·*N_ef_* ·μ this suggests an estimated effective population size of females *N_ef_* = π/2μ = 0.002/2/1.63·10^−8^ = 61,350, which is three orders of magnitude below the current estimated census population size (though ∼70% greater than inferred by Avise et al. [101]). Based on this sort of logic, Ball *et al.* (see also [101]) suggested that their data were most consistent with a recent population expansion in *A. phoeniceus*, possibly subsequent to retreat of the last ice sheets at the end of the Wisconsin Glacial. Although this argument seems reasonable, it is important to note that many factors can result in low *N_e_*/*N* ratios, including deviations from equal sex ratios, overlapping generations, and variance in reproductive success [102], all of which are likely of importance in the biology of blackbirds [100], [103], [104]. Therefore, these ratios alone may not provide compelling evidence of population expansion, and additional analysis is necessary to discover their underlying determinants.

In addition to *N_e_*/*N* estimates, among-species comparisons of effective population size estimates seem informative. The two species studied here have approximate census population sizes of at least 1.9·10^8^ for *A. phoeniceus* (based on the wintering population in the U.S.A. [80]) and 7·10^5^ for *A. tricolor* (the largest estimate from a rangewide survey [105]), a ratio of ∼250∶1. As expected given this, all of our analyses suggest that *A. phoeniceus* has a higher evolutionary effective population size than *A. tricolor* (Tables 2, 4), although our estimated ratio of polymorphism is more on the order of 3∶1. Incorporating model complexity and analytical sophistication increases neutral parameter estimates within each species, as well as the ratio of estimates between the two, from a minimum of 2.8 (π) to a maximum of 4.8 (*θ_ML_*). However, all of these esimates are vastly lower than the apparent census population size ratio of ∼250. A number of scenarios could explain this result. Perhaps the most straightforward explanation is demographic; either *A. phoeniceus* has undergone a substantial population increase or *A. tricolor* has substantially decreased. Alternatively, the two species might exhibit profound differences in female dispersal [106] or variance in reproductive success [102]; however, these latter explanations are not consistent with what is known of the biology of these species [103]–[105], [107]–[110]. Thus, this comparison also points to the importance of demographic change within *A. phoeniceus*.

An alternative explanation lies in the differential effects of selection in these two species. Either selection or population expansion is consistent with significant negative values of *F_s_* and 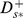 for *A. phoeniceus* [38], and positive estimates of the growth parameter *g* in analyses of this species (Table 4). A recent population bottleneck in *A. tricolor* seems unlikely given the lack of significant negative values of *D* in this species, although given the small sample size here the statistical power of the test is almost certainly an issue [111]. More compellingly, an increasing trend in polymorphism levels from south to north has been noted previously in RFLP data from *A. phoeniceus* [51], suggesting post-glacial expansion [112]. Selection on *A. phoeniceus* mtDNA does not explicitly predict such spatial structuring of diversity, although latitudinal enrichment in specific haplogroups might indicate climatic adaptation (e.g., [113], [114], but see [115]). Sampling of additional independent loci (e.g., [116], [117]) may help to untangle the relative importance of demography and selection in explaining *Agelaius* mtDNA diversity.

### Control region versus protein-coding loci as representatives of mtDNA evolution

It is clear from this discussion that the *Agelaius* control region, due to the rapid evolution of a significant subset of sites, encodes more information—particularly genealogical—than a protein-coding locus of comparable length (in this case, ND2). While true, this information comes at the cost of requiring more complex analytical machinery (including estimation of additional parameters) that may mitigate potential benefits. If mitochondrial genealogy were the primary focus of interest, it would be difficult to choose between the data sets presented here. On the one hand, the control region genealogy is more finely differentiated (i.e., the data identify more unique haplotypes); on the other hand, the ND2 genealogy twice as many well-supported nodes (eight versus four). Nevertheless, as expected due to linkage both genealogies are highly congruent and appear to reflect the same underlying history: there seems little to choose between the two on this criterion alone.

Genealogy aside, the control region and ND2 data appear to bear similar signals of selection and/or demographic change. In the first place, the power of both data sets rests on the need to estimate nuisance parameters, especially the degree of among-site rate heterogeneity (α). Even when this has been done, different analyses of these data yield varying results. For instance, accounting for among-site rate heterogeneity in Tajima's test yields significant departures from neutral equilibrium in both loci sampled from *A. phoeniceus* but not in *A. tricolor* (Table 3). Conversely, use of finite-sites models in a genealogy-based estimation of non-equilibrium dynamics for the CR suggests that *A. tricolor* has undergone a significant population expansion, whereas zero growth cannot be rejected for *A. phoeniceus* with either locus, or for the *A. tricolor* ND2 data (Table 4). Finally, only the CR data from *A. phoeniceus* showed significant departure from neutral expectation using Fu's *F_S_* (Table 3). As noted above, without additional data from other loci, it will be impossible to disentangle the effects of selection and demographic change, but both loci generally appear to bear the imprint of the same evolutionary history.

The best answer to the question of whether to prefer CR over ND2 is probably that this is the wrong question. Taken as a whole, both regions appear able to recover the underlying mitochondrial genealogy (although CR with more detail and ND2 with better support), and despite significant differences in evolutionary dynamics appear to have been influenced by similar demographic or selective effects. Given limited resources (both in technician time and sequencing dollars), a better question might be which region provides the most information per sequenced base. Assuming that we sample a single mitochondrial fragment from each individual, and that standard capillary-based Sanger sequencing yields ∼600 high quality bases per read, the first hypervariable portion of the control region clearly outperforms ND2. Sliding window analysis (using 600 base windows and 1 base step size; results not shown) of the CR data from *A. phoeniceus* reveals 45 windows having 40 polymorphic sites (maximum π = 0.012), whereas the same analysis of ND2 finds 47 windows each with a maximum of 34 polymorphic sites (maximum π = 0.008). At least five of these windows encompass seven out of the ten hypervariable CR sites identified above; additionally, they exclude the poly-pyrimidine region that makes sequencing of the complete CR challenging (see Methods). CR fragments from this region of the alignment yield resolved genealogical estimates and values for tests of neutral equilibrium similar to those obtained with the entire CR (results not shown). Future multilocus studies of blackbird genetics would do well to use this region to represent the mitochondrial genome; however, it should be noted that this result is not likely to be universal. Studies of other taxa show differing patterns of variation across the control region (e.g., [4], [25]), so it would be worthwhile to sequence the complete CR from a few individuals at the beginning of a given study to see where the majority of variation occurs, and to design primers that avoid homopolymer regions. However, we argue that one or the other hypervariable regions (or both) continue to be extremely useful in the inference of avian population history, provided that appropriate care is taken in their analysis and interpretation.

## Supporting Information

Appendix S1 **Samples analyzed in this study.**
(DOC)

Appendix S2
**Redefinition and verification of Misawa and Tajima's** 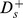 **and** 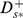 **.**
(DOC)

Appendix S3
**Code in R for calculation of** 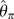 **,** 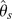 **,** 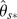 **,** 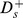 **and** 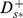 **.**
(DOC)
