# Supplementary material for: Contrasting Evolutionary Dynamics and Information Content of the Avian Mitochondrial Control Region and ND2 Gene
Source: PLoS One. 2012 Oct 5;7(10):e46403. doi: 10.1371/journal.pone.0046403 (PMC3465326; doi:10.1371/journal.pone.0046403)
Supplement: Appendix S1 — Samples analyzed in this study. (DOC) [file pone.0046403.s001.doc]

**Appendix S1**: Samples analyzed in this study. Voucher data are given in parentheses after each sample number, with collectors’ or tissue numbers in brackets where tissues reside at an institution other than the voucher or the specimen is not yet accessioned (BMNH=Bell Museum of Natural History, University of Minnesota; FMNH=Field Museum; LSUMZ=Lousiana State University Museum of Natural History; MBM=Marjorie Barrick Museum, University of Nevada, Las Vegas).

*Agelaius phoeniceus*

**USA: Minnesota (subspecies *arctolegus*), n=9**

Ap001 (BMNH [AWJ140]; Dakota Co.), Ap002 (BMNH [X7529]; Goodhue Co.), Ap003 (BMNH [MDE032]; Renville Co.), Ap004 (BMNH [X8524]; Mower Co.), Ap005 (BMNH [X8288]; Hennepin Co.), Ap006 (BMNH [MDE027]; Renville Co.), Ap007 (BMNH [X8320]; Washington Co.), Ap008 (BMNH [X7709]; Ramsey Co.), Ap009 (BMNH [MDE033]; Renville Co.)

**USA: California, Imperial County (subspecies *sonoriensis*), n=11**

Ap010 (FMNH 341961), Ap011 (FMNH 341957), Ap012 (FMNH 341956), Ap013 (FMNH 341959), Ap014 (FMNH 341954), Ap015 (FMNH 341962), Ap016 (FMNH 341958), Ap017 (FMNH 341953), Ap018 (FMNH 341955), Ap019 (FMNH 341960), Ap020 (FMNH 351160)

**USA: California, northern counties (subspecies *maillardorum*), n=3**

Ap042 (FMNH 334363; Contra Costa Co.), Ap043 (FMNH 334361; Monterey Co.), Ap044 (FMNH 334362; Monterey Co.)

**México: Puebla and Morelos, indeterminate (subspecies *gubernator* and *nelsoni*), n=7**

BB64, BB65, BB66, BB67, BB69, BB70, BB74

Note: These specimens were collected from multiple localities in two states (Morelos and Puebla) by F.C. James, for inclusion in the study of Ball et al. (1998); however, available records do not allow attribution of purified mtDNA aliquots to individuals or localities. Although particular sequences cannot be attributed to individuals, all vouchers for this material now reside in the BMNH.

**Nicaragua: Tipitapa, along shore of Lago Managua near Rio Tipitapa, n=1**

Ap041 (MBM 4319)

*Agelaius tricolor*

**USA: California, Riverside County, n=10**

At001 (LSUMZ 130808 [FMNH 7310]), At002 (FMNH 330035), At003 (LSUMZ 130801 [FMNH 7308]), At004 (LSUMZ 130796 [FMNH 7104]), At005 (FMNH 330036), At006 (FMNH 330037), At007 (LSUMZ 130833 [FMNH 7111]), At008 (LSUMZ 130809 [FMNH 7113]), At009 (LSUMZ 130832 [FMNH 7305]), At010 (LSUMZ 130807 [FMNH 7110])

*Molothrus aeneus*

**México, locality unknown, n=1**

Note: Collected by F.C. James from either Morelos or Puebla.
