# Supplementary material for: Contrasting Evolutionary Dynamics and Information Content of the Avian Mitochondrial Control Region and ND2 Gene
Source: PLoS One. 2012 Oct 5;7(10):e46403. doi: 10.1371/journal.pone.0046403 (PMC3465326; doi:10.1371/journal.pone.0046403)

**Appendix S2**: Redefinition and verification of Misawa and Tajima’s and .

Use of tests of neutral equilibrium based on measures of sequence polymorphism is widespread (e.g., [1]), although the validity of their assumptions in particular cases is seldom verified. Animal mitochondrial DNA frequently exhibits multiple substitutions per site and among-site rate heterogeneity, violating the infinite sites model underlying most of these tests (e.g., [2]). It is well known that this violation biases some tests of neutral equilibrium [3,4,5], including Tajima’s *D* [6]. Misawa and Tajima [7] proposed modified versions of Tajima’s *D* that explicitly incorporated these features, but these have not been widely applied, possibly due to an error in the equations as published. I offer a correction of the published statistics, and confirm that their critical points correspond much better to expectations under the infinite sites model than the uncorrected *D*.

Misawa and Tajima’s paper [7] expanded on results presented in Tajima [8], offering new formulae for estimating the neutral parameter *θ *= *4Nμ* (where N is the effective population size) when neutral mutation rates (*μ*) vary among sites. Estimates were given based on π, *s* and *s**, where π is the average number of nucleotide differences per site, *s* is the proportion of segregating (or polymorphic) sites, and *s** is the minimum number of mutations (the number of nucleotides at a given site minus 1) per site. As an approximate method for incorporating among-site rate heterogeneity into Tajima’s *D*, they suggested that the heterogeneity-corrected esimates of *θ* be back-translated into the units of values used in *D*. They compared the results of this procedure to expectations of *D* under the infinite sites model, and found reasonable correspondence (Table 3 and Figure 1; [7]).

Unfortunately, *s* and *s** were used inconsistently in Misawa & Tajima’s paper, which leads to confusion. In their first paragraph, they define *s* and *s** as above. This definition forms the basis of their equations 4, 5, and 6 for estimating *θ* and its variance with among-site rate heterogeneity. Confusingly, in the footnote of Table 4, they report *s* and *s** for a human mitochondrial data set as 100 and 105, which integer values must actually be *S* and *S** (i.e., the actual number of segregating sites and the minimum number of mutations, rather than those values divided by the number of sites). More importantly, they define Tajima’s *D* in their equation 7 as:

with *a_1_*, *e_1_*, and *e_2_* defined in the text analogously to Tajima (1989), and π referring to the average number of nucleotide differences *per site* and *s* the number of segregating sites *per site*. Given this, they substitute values of and *a_1_*· or *a_1_*· from their equation 5—values which are corrected for violations of the infinite sites assumption and among-site rate heterogeneity—for π and *s*, and define a new *D*, termed either or (depending on the substitution for *s*; equation 8) .

However, Tajima’s original *D* was defined in terms of (the average *number* of mutations between haplotypes—i.e., not the number per site) and *S* (the *number* of segregating sites, not the proportion), and no correction for this difference was made in the definitions of or , invalidating the published equation. Instead, each value of must be multiplied by the sequence length *L* in order to obtain a valid statistic. I redefine and as:

and ,

with *a_1_*, *e_1_*, and *e_2_* as defined in Misawa and Tajima [7]. Given the nonsensical results obtained using equation 8 as published, and comparison of simulation results reported here (see below) to table 3 and figure 1 of their paper, it is apparent that they used the correct rather than the published formulation.

In order to establish the validity and applicability of their substitution, Misawa & Tajima [7] provided data on the mean and variance of *D*, and from simulated data given varying *θ* and α (the parameter of the Γ distribution used to simulate among-site rate heterogeneity; see Table 3 of their paper). These parameter estimates indicated that the β distribution assumed for *D* rapidly became a poor fit to the empirical distribution as values of *θ* and α increased. Although the authors graphed a single simulation result (*θ *= 0.02, α = 0.1), they provided no information on the critical points of the empirical distributions, which may be of more interest than the mean and variance, given the frequent use of this procedure in hypothesis testing. In Table 1, the means, variances, and 95% and 99% critical points of empirical distributions of *D*, and (using the modified definitions presented here) are given for two values of *θ* and one α. Figure 1 presents these results graphically for *θ *= 0.02, α = 0.1, illustrating their conformity with those previously obtained (see figure 1 of [7]). These data indicate that while for these simulations approaches the expected mean and variance of β more closely than , as reported in Misawa & Tajima [7], the critical points of more closely match expectation. More extensive simulation will be required in order to establish the relative performance of these statistics, but it seems clear from results presented here and in Misawa and Tajima [7] that the proposed corrections of *D* in the presence of rate heterogeneity are both necessary and (at least over part of the relevant parameter space) effective. In Appendix S3, we present R code for calculating and given values of π, *s**, and α.

Literature Cited

1. Kreitman M (2000) Methods to detect selection in populations with applications to the human. Annual Review of Human Genetics 1: 539-559.

2. Wakely J (1993) Substitution rate variation among sites in hypervariable region 1 of human mitochondrial DNA. Journal of Molecular Evolution 37: 613-623.

3. Bertorelle G, Slatkin M (1995) The number of segregating sites in expanding human populations, with implications for estimates of demographic parameters. Molecular Biology and Evolution 12: 887-892.

4. Aris-Brosou S, Excoffier L (1996) The impact of population expansion and mutation rate heterogeneity on DNA sequence polymorphism. Molecular Biology and Evolution 13: 494-504.

5. Mes THM (2003) Demographic expansion of parasitic nematodes of livestock based on mitochondrial DNA regions that conflict with the infinite-sites model. Molecular Ecology 12: 1555-1566.

6. Tajima F (1989) Statistical method for testing the neutral mutation hypothesis by DNA polymorphism. Genetics 123: 585-595.

7. Misawa K, Tajima F (1997) Estimation of the amount of DNA polymorphism when the neutral mutation rate varies among sites. Genetics 147: 1959-1964.

8. Tajima F (1996) The amount of DNA polymorphism maintained in a finite population when the neutral mutation rate varies among sites. Genetics 143: 1457-1465.

9. Grassly NC, Harvey PH, Holmes EC (1999) Population dynamics of HIV-1 inferred from gene sequences. Genetics 151: 427-438.

10. Hey J, Wakeley J (1997) A coalescent estimator of the population recombination rate. Genetics 145: 833-846.

11. Team RDC (2011) R: A language and environment for statistical computing. R Foundation for Statistical Computing, Vienna, Austria.

Table 1. Theoretical and empirical distributions of *D*, and . Shown are the expectation, variance, 95% and 99% critical points for *D* under the infinite sites model as modeled by a β distribution [6], as well as empirical distributions of *D*, and under two simulated conditions with among-site rate heterogeneity. Data were simulated (10, 000 replicates) under a Jukes-Cantor model of sequence evolution with a discrete (10 category) approximation to the Γ distribution, using *Treevolve* 1.32 [9], and analyzed using *SITES* [10] and scripts written by the author in *R* [11].

|  |  |  |  |  | Quantiles | | | |
| --- | --- | --- | --- | --- | --- | --- | --- | --- |
| θ | α | Distribution |  |  | 0.005 | 0.025 | 0.975 | 0.995 |
| — | ∞ | *D*~β | 0.000 | 1.000 | -2.178 | -1.800 | 2.044 | 2.627 |
| 0.005 | 0.1 | *D* | 0.053 | 0.884 | -1.924 | -1.580 | 2.057 | 2.719 |
|  |  |  | -0.117 | 0.911 | -2.088 | -1.741 | 1.938 | 2.705 |
|  |  |  | -0.024 | 0.985 | -2.039 | -1.699 | 2.191 | 3.008 |
| 0.020 | 0.1 | *D* | 0.414 | 0.935 | -1.648 | -1.257 | 2.444 | 2.963 |
|  |  |  | -0.139 | 1.031 | -2.163 | -1.816 | 2.126 | 2.867 |
|  |  |  | 0.035 | 1.080 | -2.053 | -1.686 | 2.546 | 3.444 |

Figure 1. Estimated distributions of *D* and for simulated data (n=10,000, θ=0.02, α=0.1), compared with the expectation of *D* from the β distribution [6]. The critical value for the left tail of the β distribution approximation is shown.


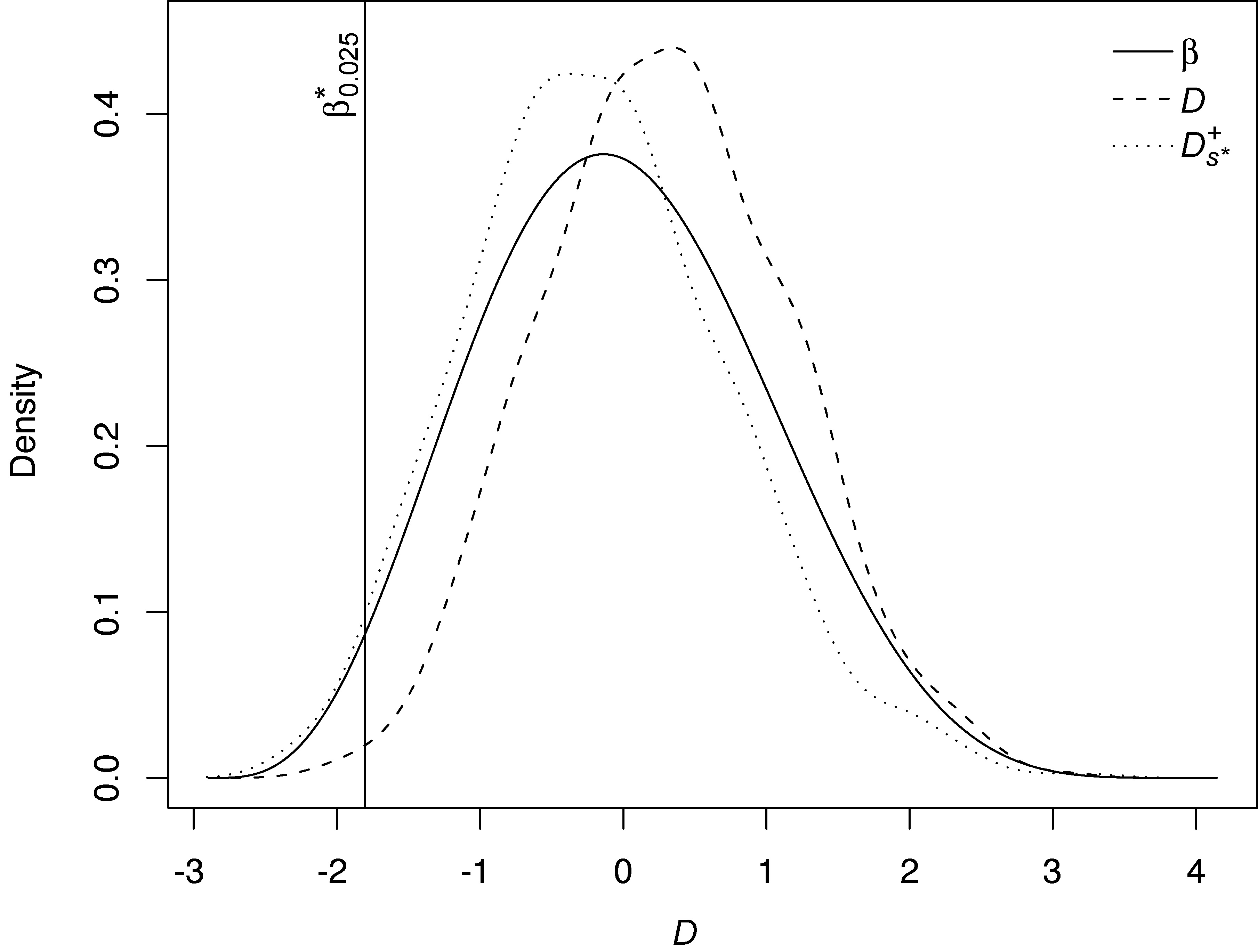

Supplement: Appendix S2 — Redefinition and verification of Misawa and Tajima's and . (DOC) [file pone.0046403.s002.doc]
