# Supplementary material for: Contrasting Evolutionary Dynamics and Information Content of the Avian Mitochondrial Control Region and ND2 Gene
Source: PLoS One. 2012 Oct 5;7(10):e46403. doi: 10.1371/journal.pone.0046403 (PMC3465326; doi:10.1371/journal.pone.0046403)
Supplement: Appendix S3 — Code in R for calculation of , , , and . (DOC) [file pone.0046403.s003.doc]

**Appendix S3**: Code in R (R Development Core Team, 2011) for calculation of $\hat{\theta}_{\pi}$,$\hat{\theta}_{s}$, $\hat{\theta}_{s*}$ (and associated standard deviations), $D_{s}^{+}$ and $D_{s*}^{+}$ (following Misawa, K. and F. Tajima 1997, *Genetics* 147: 1959-1964, as amended in Appendix S2). Depending on the function, necessary inputs are: pi=π (average pairwise sequence divergence, per site), s=*s* (proportion of segregating sites), sstar= *s** (minimum number of mutations, per site), alpha=α (parameter of the Γ distribution of among-site rate heterogeneity), n=number of sequences sampled, and L=sequence length.

thetahat.pi<-function(pi,alpha,n,L){

value<-pi*exp((pi*4*(alpha+1))/(3*alpha))

variance<-(1/(11*n^2-7*n+6))*((3*n*(n+1)*value/L)+2*(value^2)*(n^2+n+3))

return(c(value,sqrt(variance)))

}

thetahat.s<-function(s,alpha,n,L){

a1<-sum(1/(1:(n-1)))

a2<-sum(1/(1:(n-1))^2)

a3<-0.5*(a1^2-a2)

c1<-(4*a1/3)-((5*a3)/(3*a1))

value<-(s/a1)*exp((s*c1*(alpha+1))/(a1*alpha))

variance<-(1/(a1^2+a2))*((a1*value/L)+a2*value^2)

return(c(value,sqrt(variance)))

}

thetahat.sstar<-function(sstar,alpha,n,L){

a1<-sum(1/(1:(n-1)))

a2<-sum(1/(1:(n-1))^2)

a3<-0.5*(a1^2-a2)

c2<-(4*a1/3)-((7*a3)/(3*a1))

value<-(sstar/a1)*exp((sstar*c2*(alpha+1))/(a1*alpha))

variance<-(1/(a1^2+a2))*((a1*value/L)+a2*value^2)

return(c(value,sqrt(variance)))

}

Dsplus<-function(pi,s,alpha,n,L){

a1<-sum(1/(1:(n-1)))

a2<-sum(1/(1:(n-1))^2)

a3<-0.5*(a1^2-a2)

b1<-(n+1)/(3*(n-1))

b2<-2*((n^2)+n+3)/(9*n*(n-1))

c1<-(4*a1/3)-((5*a3)/(3*a1))

c3<-b1-(1/a1)

c4<-b2-((n+2)/(a1*n))+(a2/(a1^2))

e1<-c3/a1

e2<-c4/((a1^2)+a2)

thetahat.pi<-pi*exp((pi*4*(alpha+1))/(3*alpha))

thetahat.s<-(s/a1)*exp((s*c1*(alpha+1))/(a1*alpha))

numerator<-L*(thetahat.pi-thetahat.s)

denominator<-sqrt((a1*e1*L*thetahat.s)+(e2*a1*L*thetahat.s*(a1*L*thetahat.s-1)))

return(numerator/denominator)

}

Dsstarplus<-function(pi,sstar,alpha,n,L){

a1<-sum(1/(1:(n-1)))

a2<-sum(1/(1:(n-1))^2)

a3<-0.5*(a1^2-a2)

b1<-(n+1)/(3*(n-1))

b2<-2*(n^2+n+3)/(9*n*(n-1))

c2<-(4*a1/3)-((7*a3)/(3*a1))

c3<-b1-(1/a1)

c4<-b2-((n+2)/(a1*n))+(a2/a1^2)

e1<-c3/a1

e2<-c4/(a1^2+a2)

thetahat.pi<-pi*exp(pi*4*(alpha+1)/(3*alpha))

thetahat.sstar<-(sstar/a1)*exp((sstar*c2*(alpha+1))/(a1*alpha))

numerator<-L*(thetahat.pi-thetahat.sstar)

denominator<-sqrt(a1*e1*L*thetahat.sstar+e2*a1*L*thetahat.sstar*(a1*L*thetahat.sstar-1))

return(numerator/denominator)

}
